# Indents

**Published:** 1923-02

**Authors:** 


### NDENTS

**Brief Articles of Technical or Scientific Value will receive notice and comment. Contributions invited.**

---

SOME FACTS CONCERNING VULCANIZATION *as elicited by recent experiments.* When Vulcanized, Dental Gum shrinks in volume more or less, according to its composition. The average, for a number of experiments, was about 5%.

The expansion of rubber compound, when heated from 212% to the vulcanizing point, was a little less than its shrinkage in vulcanizing, averaging a little over 4%.

If the rubber is allowed to escape from the mold as it expands, the mold will be full of rubber at the *commencement* of the vulcanizing process, there will be a loss of volume during vulcanization equal to the loss of the escaping rubber by expansion, plus the shrinkage, which according to the figures shown above would be a little over 9%.

According to Dr. Gysi the total loss of volume would be from 8.5% to 14% under these conditions; depending upon the composition of the dental gum used; whether pink, red or black.

If spring pressure is applied to the flask and no outlet is provided, the flash will yield to the pressure of the expanding rubber, and the greater part, if not all of it will be retained in the mold. The rubber will diminish in volume due to its shrinkage in vulcanization, the spring pressure will cause the mold to close upon it and follow up the loss of volume by shrinkage. The mold will be practically full, at the completion of vulcanization, instead of at its beginning and the shrinkage will therefore be practically neutralized.

When the rigidly bolted flask is used, and free escape of the rubber is permitted, good results in vulcanizing are an impossibility. Spring pressure upon the flask should always be used, as this will effect the neutralization of all, or nearly all, of the shrinkage. Provision should also be made however, for compensation for the contraction of the vulcanite in cooling, and the mold should contain a small additional amount of rubber for this purpose. There will be some uncertainty to the process of vulcanizing under these circumstances if this is attempted by placing the flask in a spring clamp and inside a closed vulcanizer, although the results will be unquestionably better than if escape of the rubber is permitted. Much better results can be obtained by the use of the Compensating Vulcanizer by which means are provided for applying spring pressure at any time during the vulcanizing process and graduating it according to the judgment of the operator.

---

FAULTY EXTRACTION. During the past year, approximately 28,000 patients were examined in the dental section of the Mayo Clinic. A review of the histories of these patients disclose ample evidence that the usual method of extraction is far from adequate. Residual roots and granulomas, as well as impacted and unerupted teeth were not infrequently found in supposed edentulous sterile and aesthetic results may be obtained. As far as areas. Thirty per cent. of these patients had roots, four per cent. had impacted or unerupted teeth, and three and five-tenths per cent. had residual granulomas. With the pen view operation of extraction which is now known as alveolotomy, it is practically impossible to overlook such conditions; this is proved by post-operative roentgenograms made routinely. The literature abounds in criticism of this method, yet when it is carried out with proper restriction the results are most satisfactory.

Physicians should encourage the routine examination of teeth except in hopeless or emergency cases. Time is an important factor in emergency cases; however, the dental examination can be postponed until during convalescence after operation. The condition for which the patient is operated need not necessarily be due to mouth infection to warrant the dental examination, but the elimination of such infection is of material aid in the after-care of the patient, and in the prognosis.

Oral infection can not be eliminated by extraction alone. Treatment for pyorrhea and thorough prophylaxis are both often necessary. Great care should be exercised with regard to the number of teeth extracted at one time, and the length of time between operations. This is also true of prophylactic treatments. There should be a limited amount of scaling at the first appointment and it should not be repeated for several days.

The profession of dentistry should be discouraged from making promises to patients as to the possible result following the extraction of teeth with evidence of infection. They need the co-operation of the physician and are only responsible for the field in which they work. An intelligent report with regard to the dental findings is desired from the dentist by the physician who should take the responsibility of advising and encouraging the patient as to the outcome of the dental treatment.—Gardner, in *Journal A. D. A.*, August, 1922.

